# Integrating Point of Care Ultrasound Education into Clinical Practice at the Emergency Department

**DOI:** 10.3390/tomography8020085

**Published:** 2022-04-06

**Authors:** Kamonwon Ienghong, Lap Woon Cheung, Somsak Tiamkao, Vajarabhongsa Bhudhisawasdi, Korakot Apiratwarakul

**Affiliations:** 1 Department of Emergency Medicine, Faculty of Medicine, Khon Kaen University, Khon Kaen 40002, Thailand; kamonwan@kku.ac.th (K.I.); md221@kku.ac.th (V.B.); 2 Accident & Emergency Department, Princess Margaret Hospital, 2-10 Princess Margaret Hospital Road, Lai Chi Kok, Kowloon, Hong Kong, China; clw445@ha.org.hk; 3 Emergency Medicine Unit, Li Ka Shing Faculty of Medicine, The University of Hong Kong, Pokfulam, Hong Kong, China; 4 Department of Medicine, Faculty of Medicine, Khon Kaen University, Khon Kaen 40002, Thailand; somtia@kku.ac.th

**Keywords:** medical education, teaching, ultrasound, emergency medicine

## Abstract

Point of care ultrasound (POCUS) competency is now required learning for emergency medicine trainees. However, there is a wide range of areas that need to be assessed when determining competence. Therefore, this study aims to evaluate competence levels of POCUS skill acquisition including the areas of image acquisition, image interpretation and clinical integration of the emergency medicine residents while on shift in real clinical practice situations. This was a retrospective descriptive study. This study was conducted at Srinagarind Hospital, Thailand from January 2021 through December 2021. The data were collected and reviewed from electronic medical records, ultrasound images and video clips. All POCUS competency skills were assessed by researcher staff. Our results demonstrated that our learners had overall satisfactory competence of image acquisition, satisfactory image interpretation skills, and good clinical integration skills. However, obstetrics and gynecology (OB-GYN) ultrasound scores were poor and cardiac ultrasound had the most varied score of image quality. This study clearly showed the measurable benefits of a POCUS course being integrated into real clinical practice.

## 1. Introduction

Nowadays, point of care ultrasound (POCUS) is one of the most valuable and commonly used bedside imaging methods for emergency medicine physicians [1,2,3,4]. This is mainly due to the fact that POCUS has the ability to provide a rapid assessment of various types of emergency patients including internal medicine, surgery, trauma, pediatric, orthopedic, geriatric, pregnancy and gynecologic patients, serving to speed up the diagnostic process and is becoming increasingly more important [5,6,7].

In 2018, the Thai College of Emergency Physicians (TCEP) established a mandated program to provide POCUS training for emergency medicine residents in Thailand. The assessment of POCUS competency is now required as part of emergency medicine (EM) training. However, there is a lot of variation in how competency is measured in different emergency medicine residency programs. For example, online examinations, human models, and simulators were all used frequently as evaluation tools in previous studies [8]. This may not provide the most accurate data though as the evaluation of POCUS competency [9,10,11,12,13] mainly consists of three distinct domains: image acquisition, image interpretation, and clinical integration. According to competency based medical education [11,12,13], the development of the POCUS skills varies depending on training and practice.

In 2019, our institution implemented a mandatory POCUS training course for our emergency medicine residents. The ultrasound curriculum was a two-week rotation for first year emergency medicine residents that included a journal club (3 h), a section focused on the process of reviewing the learner’s ultrasound images (3 h), didactic lectures (300 min, each class was 30 min) in ten different content-based classes that included (1) cardiac, (2) lung, (3) abdomen, (4) aorta, (5) deep venous thrombosis, (6) soft tissue and musculoskeletal, (7) ocular, (8) kidney and urinary system, (9) obstetric and gynecologic system, (10) procedural guidance and ultrasound protocols included focused assessment with sonography for trauma (FAST) scan/extended FAST (EFAST), and finally, bedside ultrasound learning with real patients (18 h).

A previous study based in Thailand [14] demonstrated emergency medicine residents should provide POCUS knowledge in terms of POCUS used in emergency patients. To achieve competence in POCUS, emergency medicine residents must know how to use ultrasonography to detect both normal and abnormal anatomy and physiology, especially in situations most similar to actual patients’ clinical context. However, there are a limited number of studied looking at learners’ competence with POCUS in the Asia region. Thus, the aim of this study was to determine competence of POCUS skills including image acquisition, image interpretation and clinical integration of emergency residents in real clinical practice situations in the emergency department.

## 2. Materials and Methods

### 2.1. Study Design and Setting

This was a retrospective descriptive study conducted at Srinagarind Hospital, in the Department of Emergency Medicine, Khon Kaen, Thailand from 1 January 2021 to 31 December 2021. This hospital is a medical training center located in the Northeast of Thailand, which has an average of 70,000 emergency patients visiting per year.

### 2.2. Study Participants

Our study included patients who visited the emergency department throughout the 2021 year which included: (1) patients aged >18 year, (2) patients receiving POCUS examinations performed by emergency medicine residents, and (3) patients who had the POCUS video clips or images recorded by the ultrasound machines. Patients were excluded if the data were not completed to determine the ultrasound image quality and the relevance of provisional and final diagnosis.

### 2.3. Data Collection

Data collected from electronic medical records included patient characteristics, triage level according to the emergency severity index (ESI), provisional diagnosis and final diagnosis by two independent emergency physicians. The duplicate data entry was completed. If the data did not match, an emergency physician with ten years of experience with ultrasounds provided the final decision of data collection.

Ultrasound images and video clips were recorded using the ultrasound machines available in the emergency department, which included the Mindray M9 (Mindray, Shenzhen, China) and the Sonosite M turbo (Fujifilm, Washington, DC, USA) equipped with a curved array probe (1.4–5.1 MHz), phased array probe (1.1–4.4 MHz), and linear probe (3–13 MHz).

After that, POCUS competency skills were assessed by one emergency ultrasound expert who was certified with the World Interactive Network Focused On Critical UltraSound (WINFOCUS) instructor course and had one year of emergency ultrasound fellowship training, and one emergency physician who reviewed ultrasound video clips and images where they were blind to the patient’s clinical information. Then, both of them shared their opinions on the following criteria for each ultrasound video clip and image:

(1) The image acquisition skill was assessed by using a 5-point scale with the following anchors: 1 = cannot see the image, 2 = poor image quality (can see some part of the structure but cannot identify what the structure is), 3 = fair image quality (sufficient to detect what the structure is), 4 = good image quality (sufficient to visualize internal details of the structure) and 5 = excellent image quality (sufficient to demonstrate all details of the structure)
(2) The image interpretation skill was clarified as agreeing and disagreeing. The agree interpretation was defined as relevant to the sonographic findings described by the clinician who performed POCUS with the two judgement experts.
(3) The clinical integration skill was rated as relevant or irrelevant in terms of the association between the provisional diagnosis given by using ultrasound findings and the final diagnosis.

If there was a disagreement between the first two reviewers, the recorded images were subjected to a third review by a clinician with experience in ultrasound who was blinded to all clinical and sonographer information.

After that, researcher staff graded rating scores in all the competence of POCUS skill according to a previous study by Bhatnagar et al. [15] as in Table 1.

### 2.4. Study Size

The sample size was calculated based on the following formula [16]. The estimate for P was made using data from a previously published study [7], we determined that a sample size of 1024 would be required. Statistical analysis was performed with Khon Kaen University license (SPSS Inc., Chicago, IL, USA) by IBM SPSS for Windows version 27.0. Unless otherwise stated, continuous variables are reported as mean and standard deviation, and categorical variables are presented as number (n) or frequency (percent).

### 2.5. Ethical Considerations

Ethical approval was provided by the Khon Kaen University Ethics Committee for Human Research (HE641554).

## 3. Results

Over a one-year period (January to December 2021), A total of 1420 patients who received POCUS examinations were obtained for this study. Three-hundred and ninety- six patients (27.9%) had incomplete data. Thus, 1024 patients with 3014 ultrasound video clips and images were assessed. The median age of the participants was 62.5 ± 20.75 years. Males made up 61% of the sample size for this study. The majority of the patients (85.5%) were non-trauma patients. According to the emergency severity index, the majority of patients were classified as level 1 and 2 (64.3%) and level 3 (30.12%).

**Figure 1:**
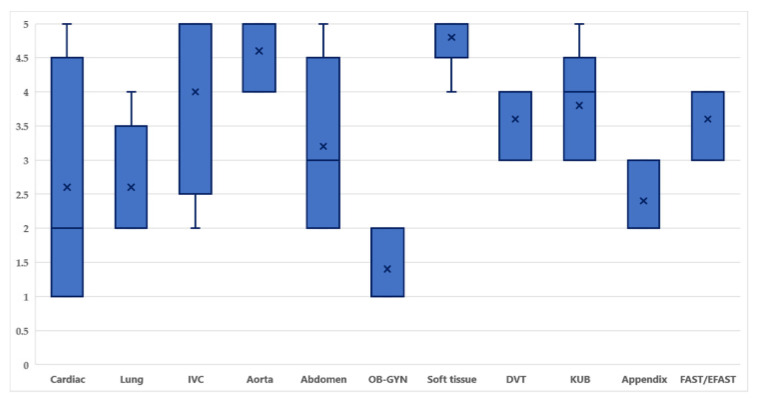
Image quality score divided by area of POCUS examination. Abbreviation: inferior vena cava (IVC), obstetrics and gynecology (OB-GYN), deep venous thrombosis (DVT), kidney and urinary bladder (KUB), and focused assessment with sonography for trauma (FAST) scan/extended FAST (EFAST).

In terms of image acquisition skills, the median image quality was 3.32 out of 5. Soft tissue images demonstrated excellent image quality, with 80% of images rated 5. OB-GYN images reported poor image quality, with 60% of images rated 1 (Figure 1). The number of images and clips contained: (1) cardiac 320 examinations, (2) lung 296 examinations, (3) inferior vena cava 304 examinations, (4) aorta 238 examinations, (5) abdomen 286 examinations, (6) OB-GYN 280 examinations, (7) soft tissue 241 examinations, (8) deep venous thrombosis (DVT) 170 examinations, (9) kidney and urinary bladder (KUB) 280 examinations, (10) appendix 298 examinations, (11) FAST/EFAST 301 examinations.

**Figure 2:**
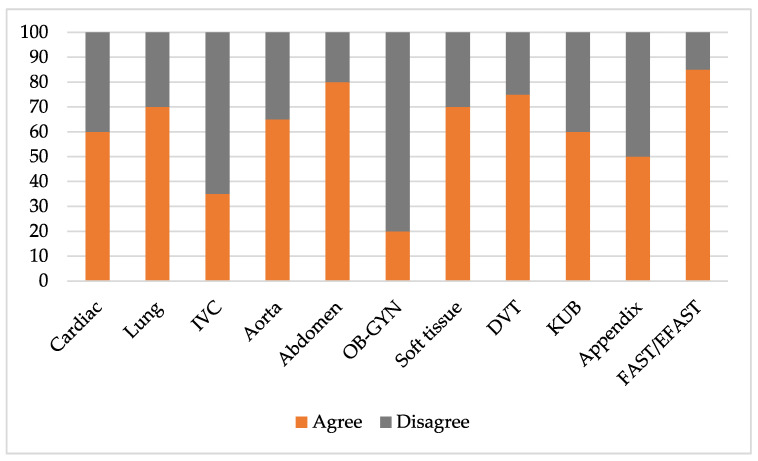
Rating scale of “Agree” and “Disagree” in the image interpretation skill divided by area of POCUS examination. Abbreviations: inferior vena cava (IVC), obstetrics and gynecology (OB-GYN), deep venous thrombosis (DVT), kidney and urinary bladder (KUB), and focused assessment with sonography for trauma (FAST) scan/extended FAST (EFAST).

In terms of the image interpretation skills, 999 out of 3014 ultrasound video clips and images were not evaluated due to poor image quality. Thus, only 2109 of the ultrasound video clips and images (69.97%) were assessed. The FAST/EFAST examination was rated as the highest percentage as 85% were rated “Agree” and the OB-GYN examination was rated as the lowest percentage with only 20% rated “Agree” (Figure 2).

In terms of the clinical integration skills, 825 out of 1024 patients (80.57%) reported the association between provisional diagnosis and final diagnosis as “relevant”.

## 4. Discussion

The aim of this study was to explore the POCUS competency skills, which included competence of image acquisition, image interpretation, and clinical integration, of emergency medicine residents throughout one year of clinical practice in the emergency department.

In terms of the competence of image acquisition; the psychomotor skill to handle probes, the visual-spatial orientation, and familiarity with the equipment are all required for POCUS. Optimizing the depth of field, image gain, and centering of the target structure to generate an image that is interpretable are additional issues for acquiring good image quality [9]. Our results indicated the overall image quality score was 3.35, which was graded as satisfactory image quality. Most residents performed POCUS well in soft tissue images, which was consistent with the previous studies [17,18] that showed healthy tissue, cellulitis, or abscesses can be recognized easily by performing POCUS. Our results also demonstrated our residents performed poorly in the OB-GYN organ system. This may be due to: (1) our residents had limited experience performing OB-GYN POCUS as they only used transabdominal ultrasonography in patients with abdominal pain and/or vaginal bleeding and a positive pregnancy test to screen for the presence of an intrauterine pregnancy (IUP) in the emergency department. It can also be noted that images collected from both transabdominal ultrasonography and transvaginal ultrasonography have ultrasound artifacts [19], which all emergency physicians who use the modality should have sufficient education and practice to be aware of these, and (2) from our previous study [14], it was noted that the frequency of this operation was less in number of times it was performed than other operations. Other results from this study showed that the competency of image acquisition for cardiac images demonstrated varied in score from 1–5. This could be explained by how inexperienced hands can misidentify gross anatomy on bedside echocardiography, the identification of anatomical structure needs to be in the hands of those with more experience [20].

In terms of the competence of image interpretation, our results showed 60.9% rated as “Agree”, which was considered to be of satisfactory level of this competency. Our residents had good competency in the area of image interpretation in FAST/EFAST examinations because this procedure contained in Thailand’s medical students curriculum. Previous studies [21,22] showed FAST/EFAST could be performed with accuracy by various levels of learners; for example, medical students, emergency medical personnel and physicians all showed high levels of competency. One study [23] compared the accuracy of image interpretation of FAST examinations between paramedics who were novice practitioners and emergency physicians that showed the accuracy of 85.6% and 87.5%, respectively. The second area in which residents had good competency of image interpretation was the abdominal area which was consistent with previous studies [24,25] that showed abdominal POCUS can be used to identify abnormal pathological states. However, the area where residents had poor image interpretation skills was the OB-GYN organ system. This may be due to the same explanations as stated previously.

In terms of the competency levels of clinical interpretation, our residents demonstrated good clinical integration skills in POCUS, with an overall accuracy of diagnosis at 80.57%. Many studies [26,27,28,29] demonstrated high levels of diagnostic accuracy using POCUS for various diseases including novel diseases, such as COVID-19. However, this study did not indicate the relevance of the diagnosis and the ultrasound finding divided into specific diseases. Because of this, this study cannot make claims to the competence of clinical interpretation in the way of a specific organ system or specific disease.

The strength of this study was that: (1) our study is the first to present substantial data on EM residents’ use of POCUS and clinical integration while actively on duty at the hospital. Despite objective structured clinical examinations (OSCEs) provided to assess multiple aspects of POCUS competency, there are limitations of clinical scenarios with simulated pathology videos or when using mannequins, which is in contrast with the real situation cases, and (2) this study included a large number of ultrasound video clips and images performed by our learners throughout one clinical practice year, which was advantageous in assessing our learners’ clinical integration. This emphasizes the need of evaluating trainees in the workplace in real time as the “core of medical competence”.

The limitations of this study were: (1) This study was not designed to demonstrate the limitation of POCUS skills in individuals as learners; (2) This study focused on the overall POCUS skills of our emergency medicine residents after finishing our ultrasound training, which may not be generalizable to other specialties; (3) Although our result demonstrating the percentage of satisfactory score was 61% that seems a very low score for ‘satisfactory’. However, compared with the previous study [15], which studied the measurement score of OSCEs skill, our results demonstrated the same satisfactory level; and (4) In terms of the scoring of ‘image quality’, which has a confounding factor, in that some patients are much more difficult, and that clinical diagnosis may not be dependent upon images but on the real-time assessment.

## 5. Conclusions

This study identified the competence of POCUS skill after completing our ultrasound training, which demonstrated overall satisfactory image quality scores, satisfactory image interpretation skills and good clinical integration skills of our learners. However, the OB-GYN score was poor, and cardiac ultrasound was the most varied score in relation to image quality. Presently, we hope this study will emphasize the assessment of POCUS competency of residency training, especially in the area of point-of-care ultrasound clinical integration.

## 6. Future Work

POCUS education is still faced with many limitations in our country. One of the potential avenues to develop quality POCUS education programs is by implementing the formal training of POCUS, creating a formal assessment tool of POCUS competency, and integrating POCUS education into real clinical practice. Future work of POCUS education should be focused on long-term measures of student performance and the utility of POCUS used in specific disease cases.

## Figures and Tables

**Table 1:** Score interpretation and grading of the performance of competency level.

Score (Percentage)	Grade	Interpretation
>70	Good	Comfortable and familiar with the tasks expected to perform
60–69	Satisfactory	Familiar with the tasks Needs occasional checks
40–59	Need improvements	Needs supervision for some tasks, guidance for others Expected to improve by self- study
<40	Unsatisfactory performance	Needs close supervision at work

## Data Availability

The data presented in this study are available on request from the corresponding author.
